# Genetic Resistance Determinants, *In Vitro* Time-Kill Curve Analysis and Pharmacodynamic Functions for the Novel Topoisomerase II Inhibitor ETX0914 (AZD0914) in *Neisseria gonorrhoeae*

**DOI:** 10.3389/fmicb.2015.01377

**Published:** 2015-12-10

**Authors:** Sunniva Foerster, Daniel Golparian, Susanne Jacobsson, Lucy J. Hathaway, Nicola Low, William M. Shafer, Christian L. Althaus, Magnus Unemo

**Affiliations:** ^1^Institute for Infectious Diseases, University of BernBern, Switzerland; ^2^Institute of Social and Preventive Medicine, University of BernBern, Switzerland; ^3^WHO Collaborating Centre for Gonorrhoea and other STIs, National Reference Laboratory for Pathogenic Neisseria, Faculty of Medicine and Health, Örebro UniversityÖrebro, Sweden; ^4^Department of Microbiology and Immunology, Emory University School of Medicine, AtlantaGA, USA; ^5^Laboratories of Bacterial Pathogenesis, Veterans Affairs Medical Center, DecaturGA, USA

**Keywords:** gonorrhea, treatment, antimicrobial resistance, time-kill curve analysis, pharmacodynamics, DNA topoisomerase II inhibitor, ETX0914

## Abstract

Resistance in *Neisseria gonorrhoeae* to all available therapeutic antimicrobials has emerged and new efficacious drugs for treatment of gonorrhea are essential. The topoisomerase II inhibitor ETX0914 (also known as AZD0914) is a new spiropyrimidinetrione antimicrobial that has different mechanisms of action from all previous and current gonorrhea treatment options. In this study, the *N. gonorrhoeae* resistance determinants for ETX0914 were further described and the effects of ETX0914 on the growth of *N. gonorrhoeae* (ETX0914 wild type, single step selected resistant mutants, and eﬄux pump mutants) were examined in a novel *in vitro* time-kill curve analysis to estimate pharmacodynamic parameters of the new antimicrobial. For comparison, ciprofloxacin, azithromycin, ceftriaxone, and tetracycline were also examined (separately and in combination with ETX0914). ETX0914 was rapidly bactericidal for all wild type strains and had similar pharmacodynamic properties to ciprofloxacin. All selected resistant mutants contained mutations in amino acid codons D429 or K450 of GyrB and inactivation of the MtrCDE eﬄux pump fully restored the susceptibility to ETX0914. ETX0914 alone and in combination with azithromycin and ceftriaxone was highly effective against *N. gonorrhoeae* and synergistic interaction with ciprofloxacin, particularly for ETX0914-resistant mutants, was found. ETX0914, monotherapy or in combination with azithromycin (to cover additional sexually transmitted infections), should be considered for phase III clinical trials and future gonorrhea treatment.

## Introduction

The sexually transmitted infection gonorrhea, caused by *Neisseria gonorrhoeae*, is a significant global public health concern (World Health Organization [WHO], 2008; Centers for Disease Control and Prevention [CDC], 2012; World Health Organization and Department of Reproductive Health and Research, 2012a,b). *In vitro* resistance, including high-level resistance, to the last available option for first-line empirical antimicrobial monotherapy, the third-generation extended-spectrum cephalosporin ceftriaxone, has emerged (Ohnishi et al., 2011; Cámara et al., 2012; Ison, 2012; Unemo and Nicholas, 2012; Unemo et al., 2012a; Unemo and Shafer, 2014). Sporadic failures to treat pharyngeal gonorrhea with ceftriaxone have also been verified in several countries (Ohnishi et al., 2011; Unemo and Nicholas, 2012; Unemo et al., 2011, 2012b; Chen et al., 2013; Read et al., 2013; Golparian et al., 2014a).

New drugs with mechanisms of action that are distinct from previously used antimicrobials are urgently needed to forestall scenarios in which gonorrhea becomes very difficult to treat or even untreatable. Spiropyrimidinetriones represent a recently developed class of antimicrobials that inhibit bacterial DNA type II topoisomerases, i.e., DNA gyrase and topoisomerase IV (Basarab et al., 2015). Their mechanisms of action differs from that of all other available antimicrobials, including DNA topoisomerase inhibitors such as the fluoroquinolones (Mayer and Janin, 2014; Huband et al., 2015; Kern et al., 2015). ETX0914 (also known as AZD0914; Entasis Therapeutics, Waltham, MA, USA) is a novel, not yet clinically used or commercially available, spiropyrimidinetrione, aimed for oral administration, with potent *in vitro* activity against key Gram-positive, fastidious Gram-negative, atypical and anaerobic bacterial species, including isolates with high-level resistance to fluoroquinolones (Huband et al., 2015). ETX0914 was recently shown to have an excellent *in vitro* activity against a wide variety of clinical *N. gonorrhoeae* strains, including many multidrug-resistant (MDR) and extensively drug-resistant (XDR) strains. The minimum inhibitory concentration (MIC) values for ETX0914 ranged between ≤0.004 and 0.25 mg/L and no cross-resistance to the fluoroquinolone ciprofloxacin or other antimicrobials previously or currently used in the treatment of gonorrhea was observed (Jacobsson et al., 2014).

ETX0914 stabilizes and arrests the cleaved covalent complex of DNA gyrase with double-strand broken DNA and thus blocks religation of the double-strand cleaved DNA to form fused circular DNA (Mayer and Janin, 2014; Huband et al., 2015; Kern et al., 2015). Accordingly, ETX0914 inhibits DNA biosynthesis and replication by an accumulation of DNA double strand cleavages and prevention of religation, in contrast to the fluoroquinolones (Mayer and Janin, 2014; Palmer et al., 2014; Huband et al., 2015; Kern et al., 2015). In *N. gonorrhoeae*, resistance to ETX0914 has been selected *in vitro* in a few gonococcal strains and the resistance mutations D429N and/or K450T, occurring at low frequencies, were mapped to *gyrB* (Alm et al., 2015). The effects on the MIC of ETX0914 by other *gyrB* mutations and possible mutations in *gyrA*, *parC*, *parE*, or *porB*, encoding the PorB porin, genes as well as over-expression of eﬄux pumps remain unknown.

The evaluation of a new antimicrobial includes an assessment of the relationship between exposure to the drug and bacterial growth (Drusano, 2004). The MIC gives a biological measure of the susceptibility of a pathogen to an antimicrobial, but does not give any information about how different antimicrobial concentrations affect bacterial growth over time (Li et al., 1999). Regoes et al. (2004) introduced the concept of pharmacodynamic functions, which are related to maximum effect (*E*_max_) models, to examine the relationship between antimicrobial concentration and bacterial growth and death rates, estimated from *in vitro* time-kill curves. Antimicrobials with identical MICs but a different pharmacodynamic function can have differences in antimicrobial efficacy. Appropriate *in vitro* growth and pharmacodynamic assays for the obligate human pathogen *N. gonorrhoeae* have been lacking and would be exceedingly valuable.

The aims of the present study were to extend the characterization of the resistance determinants for ETX0914 and to investigate how ETX0914 affects the growth of *N. gonorrhoeae* (ETX0914 wild type, single step resistant mutants and eﬄux pump mutants) using a novel *in vitro* time-kill curve analysis to estimate pharmacodynamic parameters of the new antimicrobial (Foerster et al., 2015). The results for ETX0194 were compared to those of ciprofloxacin, azithromycin, ceftriaxone, and tetracycline. Combinations of these antimicrobials with ETX0194 were also further investigated using checkerboard analysis and time-kill curve analysis.

## Material and Methods

### Bacterial Strains

The international *N. gonorrhoeae* reference strains WHO F, WHO O, WHO P (Unemo et al., 2009), the ceftriaxone high-level resistant XDR strain H041 (Ohnishi et al., 2011), and single step selected resistant mutants of WHO O and P were included in this study. The relevant phenotypic and genetic characteristics of these parental strains are summarized in **Table 1**. All strains were cultured on GCAGP agar plates (3.6% Difco GC Medium Base agar [BD, Diagnostics, Sparks, MD, USA] supplemented with 1% hemoglobin [BD, Diagnostics], 1% IsoVitalex [BD, Diagnostics] and 10% horse serum) for 18–20 h at 37°C in a humid 5% CO_2_-enriched atmosphere.

**Table 1:** Relevant phenotypic and genetic characteristics of *Neisseria gonorrhoeae* strains examined.

Strain characteristics	WHO F	WHO O	WHO P	H041
ETX0914 (MIC, mg/L)	0.064	0.125	0.25	0.125
Ciprofloxacin (MIC, mg/L)	0.004	0.008	0.004	>32
Azithromycin (MIC, mg/L)	0.125	0.25	2	1
Ceftriaxone (MIC, mg/L)	<0.002	0.032	0.032	4
Tetracycline (MIC, mg/L)	0.25	1	0.5	4
*gyrA* codon 91, 95	WT	WT	WT	S91F, D95N
*gyrB* codon 429	WT	WT	WT	WT
*mtrR* promoter region 13 bp inverted repeat	WT	Deletion of A	A→C substitution	deletion of A
*mtrR* coding region frame-shift mutation	—	—	T insert at bp 60	–
*porB1b* codon 120	NA	G120K	WT	G120K
*porB1b* codon 121	NA	A121D	A121D	A121D
NG-MAST ST	ST3303	ST495	ST3305	ST4220
MLST	ST10934	ST1902	ST8127	ST7363


*MIC, minimum inhibitory concentration; WT, wild type; NA, not applicable; NG-MAST, *N. gonorrhoeae* multiantigen sequence typing; ST, sequence type; MLST, multi-locus sequence typing. Data from previous publications (Unemo et al., 2009; Ohnishi et al., 2011) also included.*

### Antimicrobial Susceptibility Testing

The MICs (mg/L) of ETX0914 (Entasis Therapeutics, Waltham, MA, USA) were determined by agar dilution technique, in accordance with the Clinical and Laboratory Standards Institute (CLSI) guidelines (Clinical and Laboratory Standards Institute, 2014) and as previously described (Jacobsson et al., 2014). Susceptibility to ciprofloxacin, azithromycin, ceftriaxone, and tetracycline was determined using the Etest method (bioMérieux, Marcy l’Etoile, France) in accordance with the manufacturer’s instructions, on GCRAP agar plates (3.6% Difco GC Medium Base agar [BD, Diagnostics] supplemented with 1% hemoglobin [BD, Diagnostics] and 1% IsoVitalex [BD, Diagnostics]).

### Selection of Single Step ETX0914-Resistant Mutants

Resistant mutants were selected on GC agar plates (3.6% Difco GC Medium Base agar [BD, Diagnostics] supplemented with 1% IsoVitalex [BD, Diagnostics]) containing 2-fold increasing concentrations of ETX0914 (0.032-8 mg/L). Briefly, WHO F, WHO P, WHO O, and H041 (**Table 1**) were cultured on GCAGP agar plates for 18–20 h at 37°C in a humid 5% CO_2_-enriched atmosphere. Fresh cultures (18 h) from 10 agar plates were pooled and suspended in 2 mL of sterile PBS. A dilution series (ranging from 1:100 to 1:10^14^) of the bacterial suspension in PBS was plated on compound free GC agar plates. Undiluted 100 μL aliquots were plated on ETX0914-containing GC agar plates and grown for 48 h at 37°C in a humid 5% CO_2_-enriched atmosphere. Selected resistant mutants that grew at the highest ETX0914 concentration were sub-cultured for 24 h on GCAGP agar plates and frozen at -70°C.

### *gyrB* Sequencing

To identify the ETX0914 resistance mutations in the selected mutants, *gyrB* was amplified by PCR and sequenced as described (Jacobsson et al., 2014).

### Generation of Eﬄux Pump Mutants

The *mtrD*, *macA*, and *norM* genes, coding for components of the MtrCDE, MacAB and NorM eﬄux pumps, were inactivated in strains by spot transformation (Gunn and Stein, 1996) using 0.1 μg of chromosomal DNA from genetic derivatives of strain FA19 or wild type strain FA19 bearing insertions in *mtrD* (strain KH14; *mtrD::kan*), *macA* (strain BR54 *macA::spc*) or *norM* (strain FA19 *norM::kan*), as previously described (Golparian et al., 2014b).

### Time-kill Experiments

Time-kill curve analyses were performed by culturing the *N. gonorrhoeae* strains in liquid Graver–Wade (GW) medium (Wade and Graver, 2007). Eleven antimicrobial-concentrations in doubling dilutions ranging from 0.016 × MIC to 16 × MIC, in Sarstedt 96-well round bottom microtiter plates (360 μL wells) were examined for the following antimicrobials: ETX0914 (Entasis Therapeutics), ciprofloxacin (Sigma Aldrich, USA), azithromycin (Sigma Aldrich), ceftriaxone (Sigma Aldrich), and tetracycline (Sigma Aldrich). Growth curves were initially performed to confirm that all strains would reach a stable early- to mid-log phase after 4 h of pre-incubation in antimicrobial-free GW medium. Subsequently, a 0.5 McFarland inoculum of *N. gonorrhoeae* was prepared in sterile PBS from cultures grown on GCAGP agar plates for 18–20 h at 37°C in a humid 5% CO_2_-enriched atmosphere. For each strain, 30 μL of the inoculum was diluted in 15 mL pre-warmed antimicrobial-free GW medium (37°C) and 90 μL per well was dispersed in Sarstedt 96-well round bottom microtiter plates (360 μL wells). The plates were pre-incubated for 4 h shaking at 150 rpm, 35°C in a humid 5% CO_2_-enriched atmosphere. To each well containing 90 μL of pre-incubated bacteria, 10 μL of one of the antimicrobial concentrations (or PBS) was added, resulting in eight identical rows containing bacteria exposed to 11 different antimicrobial concentrations and one untreated control. Colony forming units (CFUs) were measured using a modified Miles and Misra method as previously described (Chen et al., 2003). At 0, 1, 2, 3, 4, 5, and 6 h after exposure, the total volume of one row was removed using a multichannel pipette and diluted in PBS in six subsequent 1:10 dilutions. Ten μL droplets of each dilution were spotted on GCRAP agar plates (diameter 14 cm; pre-dried with open lid in a sterile environment for 30–60 min). After drying the plates (∼5–10 min), they were incubated for 24 h at 37°C in a humid 5% CO_2_–enriched atmosphere. Colonies were then counted and numbers extrapolated to CFUs/mL by multiplying the initial volume of 10 μL with 100 and the dilution factor.

### Pharmacodynamic Function and Hierarchical Clustering

The results of the novel *in vitro* time-kill curve analysis were used to estimate pharmacodynamic parameters of antimicrobials. In brief, the bacterial net growth rates were estimated from changes in the density of viable bacteria (CFU/mL) between 0 and 6 h of the time-kill experiments, as previously described (Foerster et al., 2015). A pharmacodynamic function was then fitted to the bacterial net growth rates in response to different concentrations of antimicrobials as earlier detailed (Regoes et al., 2004). In this pharmacodynamic function, the bottom asymptote (parameter ψ_min_) and top asymptote (parameter ψ_max_) represent the minimal and maximal bacterial net growth rate at high concentrations and in absence of antimicrobial, respectively. The slope of the sigmoidal curve at the inflection point (Hill coefficient or parameter κ) describes the steepness of the relationship between bacterial growth and antimicrobial concentration. The pharmacodynamic MIC (parameter zMIC) is the antimicrobial concentration that results in a net growth rate of zero. The heatmap was produced by plotting the numeric values of the minimal bacterial net growth rate at high concentrations of antimicrobial (ψ_min_) and the Hill coefficient *(κ*; steepness of the relationship between bacterial growth and antimicrobial concentration) in a color gradient from red (high values) to blue (low values). The fitting routine was based on self-starter models as implemented in the R package drc (Ritz and Streibig, 2005). The hierarchical clustering was performed using the complete linkage algorithm as implemented in the package heatmap3 (Zhao et al., 2014). The distances in the resulting dendrogram represented similarities between strains and antimicrobials. Further details regarding fitting the pharmacodynamic function to the bacterial net growth rates in response to different concentrations of antimicrobials and all estimated parameters using the function in the present study have been summarized in **Supplementary Table S1**.

### Checkerboard Assay

Checkerboard assays for examination of antimicrobial combinations were performed as previously described (Meletiadis et al., 2010), with minor modifications. Doubling dilution series for both antimicrobials starting at 16 × MIC of each antimicrobial (combined in 1:1 ratios) were prepared in PBS. Five micro liter of each of the dilution series were combined in a Sarstedt 96 well round bottom microtiter plate (360 μL wells), resulting in a gradient that contained the highest concentration of drugs in well A1. A bacterial suspension in GW medium (Wade and Graver, 2007), containing approximately 10^5^ CFU/mL from 18–20 h old culture on GCAGP agar plates was prepared and incubated for 4 h at 150 rpm, 37°C, humid 5% CO_2_-enriched atmosphere in a 50 mL Erlenmeyer flask. These cultures were added to the checkerboard plates containing the antimicrobial combinations (90 μL culture + 10 μL antimicrobial dilution per well). Bacterial growth in each well was assessed visually after 24 h of incubation with a magnifying glass. The isoeffective combinations were determined as the lowest drug concentrations showing no visual growth (optically clear well).

### Fractional Inhibitory Concentration Index (FICI) Analysis

The fractional inhibitory concentration index (FICI) was calculated as follows (White et al., 1996):

$$FICI=FIC_{A}+FIC_{B}=MIC_{A}^{comb}/MIC_{A}^{alone}+MIC_{B}^{comb}/MIC_{B}^{alone}$$

Among all the FICIs calculated for all isoeffective combinations, the minimum FIC (FICI_min_) and the maximum FIC (FICI_max_) were reported in order to identify synergistic and antagonistic interactions. Cut-off criteria of ≤0.5 for synergy and >4 for antagonism were applied (Odds, 2003). The FICI was also calculated for the zMIC values of the time-kill experiments, applying the same criteria. To calculate the concentrations of both antimicrobials in the combination, the ratio was calculated dividing zMIC/(maximal concentration A + maximal concentration of B). The antimicrobial interaction was only considered to be verified if checkerboard and time-kill assay results agreed.

## Results

### Selection of Single Step ETX0914-Resistant Mutants

Single step mutants were selected at very low frequencies on GC agar plates (<1.9 × 10^-14^) from the reference strains WHO F, O, and P, but not for H041. All selected resistant mutants contained a single amino acid alteration (D429N, D429A, or K450T) in GyrB, which resulted in ETX0914 MICs of 0.5–4 mg/L. However, these *gyrB* mutations did not significantly affect the MICs of ciprofloxacin, azithromycin, ceftriaxone, and tetracycline (**Table 2**). The selected resistant mutants OM-5 and PM-4 were selected for further analysis.

**Table 2:** Single step selected resistant mutants, *gyrB* mutations, frequency of selected mutations, and effects on MIC of examined antimicrobials.

Isolate	Description	*gyrB* mutation	Frequency of mutation (CFU/mL)^a^	MIC (mg/L)^b^				
	
				AZM	ETX0914	CIP	CRO	TET
WHO F	WT	WT		0.125	0.064	0.004	0.004	0.25
FM-1	First step mutant	D429N	<3 × 10^-14c^	0.125	0.5	0.008	<0.002	0.5
FM-3	First step mutant	D429N	<3 × 10^-14c^	0.125	0.5	0.008	<0.002	0.25
FM-5	First step mutant	D429N	<3 × 10^-14c^	0.125	0.5	0.008	<0.002	0.25
FM-6	First step mutant	D429N	<3 × 10^-14c^	0.125	0.5	0.008	<0.002	0.25
FM-7	First step mutant	D429N	<3 × 10^-14c^	0.125	0.5	0.016	<0.002	0.5
FM-8	First step mutant	D429N	<3 × 10^-14c^	0.125	0.5	0.008	<0.002	0.5
WHO O	WT	WT		0.25	0.125	0.008	0.032	1
OM-1	First step mutant	D429N	<3 × 10^-14c^	0.5	1	0.032	0.016	2
OM-2	First step mutant	D429N	<3 × 10^-14c^	0.5	1	0.032	0.016	2
OM-3	First step mutant	D429N	<3 × 10^-14c^	0.5	1	0.032	0.016	2
OM-4	First step mutant	D429N	<3 × 10^-14c^	0.5	1	0.032	0.016	2
OM-5	First step mutant	D429N	<3 × 10^-14c^	0.5	1	0.032	0.032	2
OM-6	First step mutant	D429N	<3 × 10^-14c^	0.5	0.5	0.032	0.016	2
WHO P	WT	WT		2	0.25	0.004	0.004	0.5
PM-1	First step mutant	D429N	2 × 10^-14^	4	2	0.008	0.004	1
PM-2	First step mutant	D429N	2 × 10^-14^	4	4	0.032	0.008	1
PM-3	First step mutant	D429N	2 × 10^-14^	4	2	0.008	0.004	1
PM-4	First step mutant	D429N	2 × 10^-14^	4	4	0.032	0.004	1
PM-5	First step mutant	D429A	2 × 10^-14^	4	1	0.008	0.004	2
PM-6	First step mutant	D429N	2 × 10^-14^	4	2	0.008	0.008	2
PM-7	First step mutant	D429N	2 × 10^-14^	4	2	0.008	0.008	2
PM-8	First step mutant	D429N	2 × 10^-14^	2	2	0.004	0.004	1
PM-9	First step mutant	K450T	2 × 10^-14^	4	2	0.008	0.008	1
PM-10	First step mutant	D429N	2 × 10^-14^	8	2	0.008	0.008	1
H041	Parent	WT		1	0.125	>32	8	4
H041-1	No mutant^a^	–	–	–	–	–	–	-
H041-2	No mutant^a^	–	–	–	–	–	–	–
H041-3	No mutant^a^	–	–	–	–	–	–	–
H041-4	No mutant^a^	–	–	–	–	–	–	–
H041-5	No mutant^a^	–	–	–	–	–	–	–
H041-6	No mutant^a^	–	–	–	–	–	–	–


*^a^Using a more nutritious agar medium (GCAGP agar plates, including also 1% hemoglobin and 10% horse serum) and 72 h culture selected resistant mutants in all four strains, that is, with a frequency of 8 × 10^-*9*^ for H041, 5 × 10^-*9*^ for WHO F, and 1 × 10^-*8*^ for WHO P and WHO O. ^b^Only whole MIC doubling dilutions from the Etest reported. ^c^Frequency below limit of detection, i.e., numbers of CFUs that could be counted. AZM, azithromycin; CIP, ciprofloxacin; CRO, ceftriaxone; TET, tetracycline; WT, wild type.*

### Effect on ETX0914 Susceptibility by Inactivation of Eﬄux Pumps

By inactivation of the MtrCDE eﬄux pump, susceptibility to ETX0914 was fully restored (MIC = 0.125 mg/L) in both resistant mutants and the ETX0914 MIC values in the ETX0914 wild type WHO F, O, P and H041 strains decreased significantly (3- to 5-fold). Additional inactivation of the MacAB or NorM eﬄux pumps decreased the MIC of ETX0914 in the H041 strain (from 0.125 mg/L to 0.008 mg/L), but these inactivations had no significant effect on the MICs of ETX0914 in the other strains. As previously shown (Golparian et al., 2014b), the inactivation of the MtrCDE eﬄux pump significantly increased the susceptibility also to azithromycin in all strains and to ceftriaxone in most strains (**Table 3**).

**Table 3:** Minimum inhibitory concentration (MICs)^a,b^ in *Neisseria gonorrhoeae* wild type strains and selected ETX0914-resistant mutants after inactivation of the MtrCDE, MacAB, and NorM eﬄux pumps.

Bacterial strain	ETX0914	CIP	AZM	CRO	TET
WHO O WT	0.125	0.008	0.25	0.032	1
MtrCDE IA	0.008	0.004	0.064	0.004	0.5
MacAB IA	0.125	0.008	0.5	0.032	2
NorM IA	0.125	0.008	0.5	0.032	2
OM-5	1	0.032	0.5	0.064	2
MtrCDE IA	0.125	0.008	0.064	0.008	1
MacAB IA	1	0.032	0.5	0.064	2
NorM IA	1	0.032	0.5	0.032	2
WHO P WT	0.25	0.004	2	0.004	0.5
MtrCDE IA	0.008	0.004	0.064	0.004	0.25
MacAB IA	0.25	0.008	4	0.008	1
NorM IA	0.25	0.008	4	0.008	2
PM-4	4	0.016	4	0.008	1
MtrCDE IA	0.125	0.008	0.064	0.004	1
MacAB IA	4	0.016	2	0.008	1
NorM IA	4	0.016	2	0.008	2
WHO F WT	0.064	0.004	0.125	<0.002	0.25
MtrCDE IA	0.008	0.004	0.064	<0.002	0.25
MacAB IA	0.064	0.004	0.125	<0.002	0.25
NorM IA	0.064	0.004	0.25	<0.002	1
H041 WT	0.125	>32	1	4	2
MtrCDE IA	0.004	16	0.064	1	1
MacAB IA	0.008	>32	0.25	2	0.5
NorM IA	0.008	>32	0.5	2	4


*AZM, azithromycin; CIP, ciprofloxacin; CRO, ceftriaxone; TET, tetracycline; WT, wild type; IA, Inactivation. ^a^Only whole MIC doubling dilutions in mg/L from the Etest reported. ^b^Data from previous publication (Golparian et al., 2014b) also included.*

### Time-kill Curve Analysis

**FIGURE 1:**
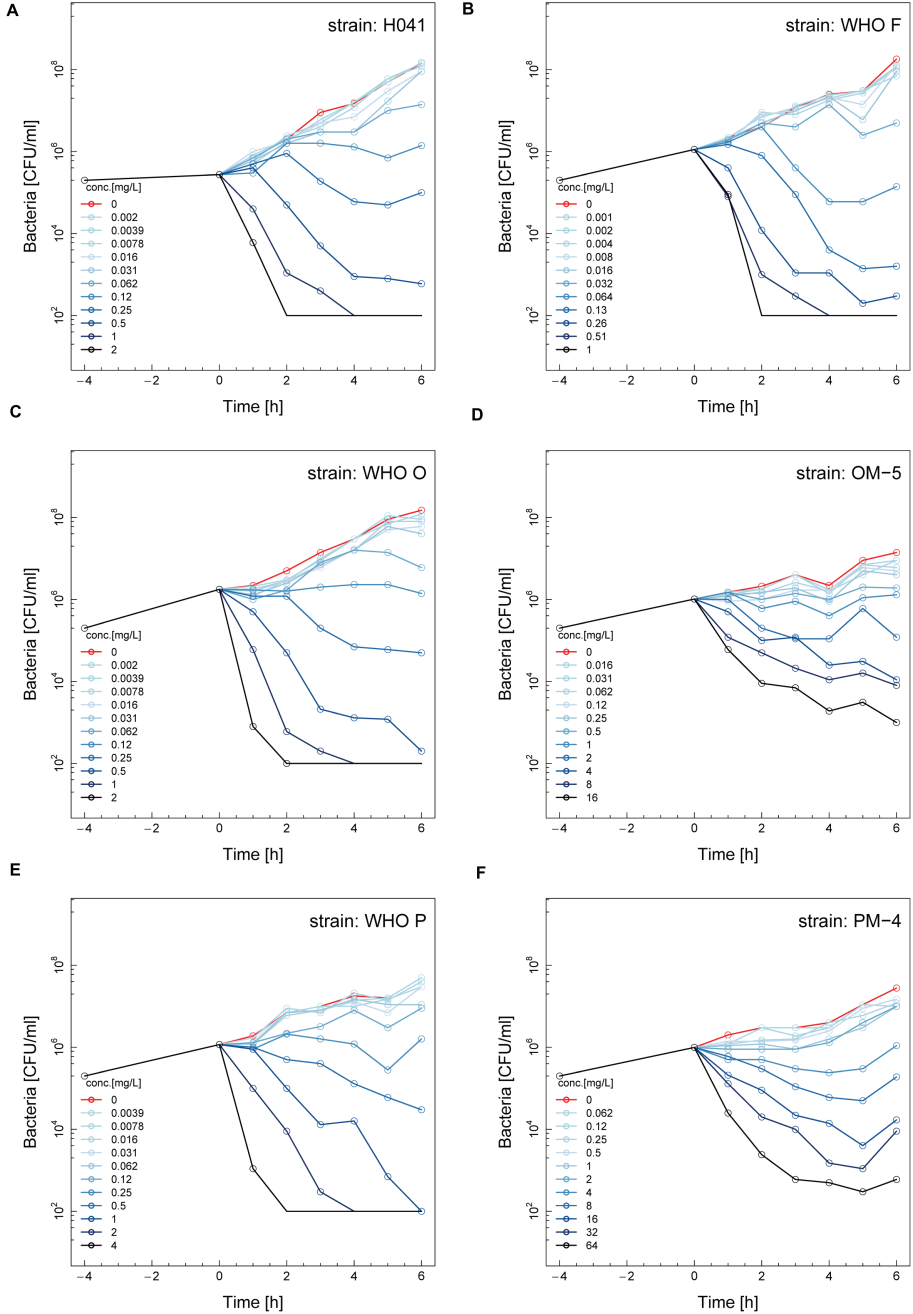
**Time-kill curves of six different *Neisseria gonorrhoeae* strains with ETX0914: H041 **(A)**, WHO F **(B)**, WHO O **(C)**, OM-5 (**D**; *in vitro* selected resistant mutant from WHO O), WHO P **(E)**, and PM-4 (**F**; *in vitro* selected resistant mutant from WHO P).** Untreated controls are shown in red, each line corresponds to a doubling concentration of the antimicrobial, and circles indicate each measured time point. The limit of detection in the assay was 100 colony forming units (CFUs)/mL.

The phenotypic effects of ETX0914 on the ETX0914 wild type WHO F, O, P and H041 strains, as well as the resistant OM-5 and PM-4 mutants were assessed in time-kill curve analysis. Similar time-kill curve profiles were obtained for all the wild type strains (**Figures 1A–C,E**). The CFUs of all wild type strains were reduced to below the limit of detection (100 CFU/mL) at 8 × MIC and 16 × MIC, and killed, albeit more slowly at 2 × MIC and 4 × MIC, within 4 h of exposure. The net growth rates decreased quickly in the first hour and then leveled off. ETX0914 also killed the resistant mutants OM-5 and PM-4 at concentrations above the MIC but the mutants were above the limit of detection (100 CFU/mL) even at the highest ETX0914 concentration (16 × MIC) for the exposure time of 6 h. The decrease in net growth rates in response to increased ETX0914 concentrations was smaller and more gradual compared to their respective wild type strains (**Figures 1D,F**).

### Pharmacodynamic Functions

**FIGURE 2:**
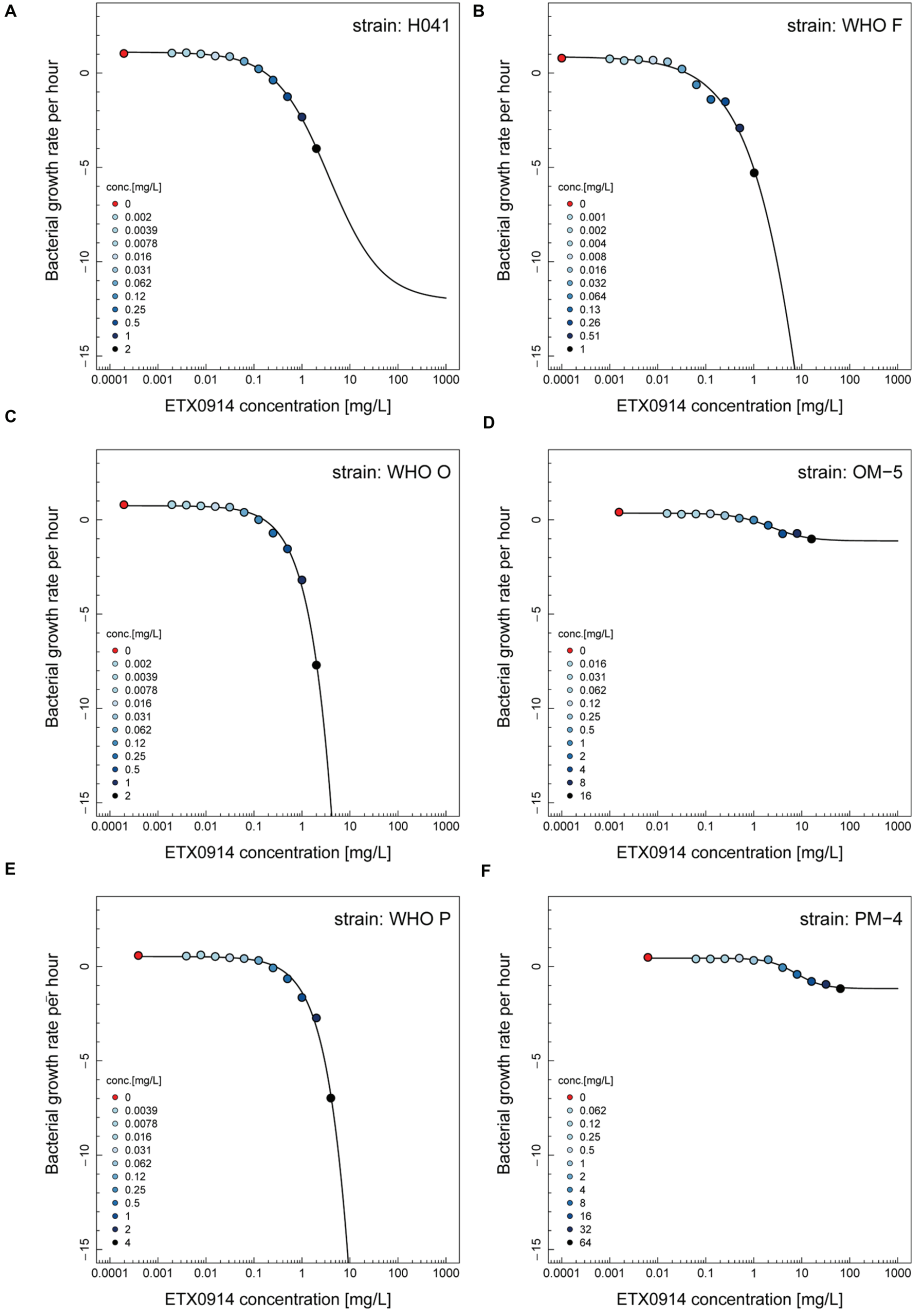
**Pharmacodynamic functions based on the time-kill curve data for ETX0914 and six different *Neisseria gonorrhoeae* strains: H041 **(A)**, WHO F **(B)**, WHO O **(C)**, OM-5 (**D**; *in vitro* selected resistant mutant from WHO O), WHO P **(E)**, and PM-4 (**F**; *in vitro* selected resistant mutant from WHO P).** The bacterial growth rates per hour derived from linear regression on time-kill curves for each ETX0914 concentration are plotted. Growth in absence of ETX0914 was plotted as a red circle, at a tenth of the lowest concentration to allow plotting on a log scale, and decreasing ETX0914 concentrations are shown from dark to light blue color.

**FIGURE 3:**
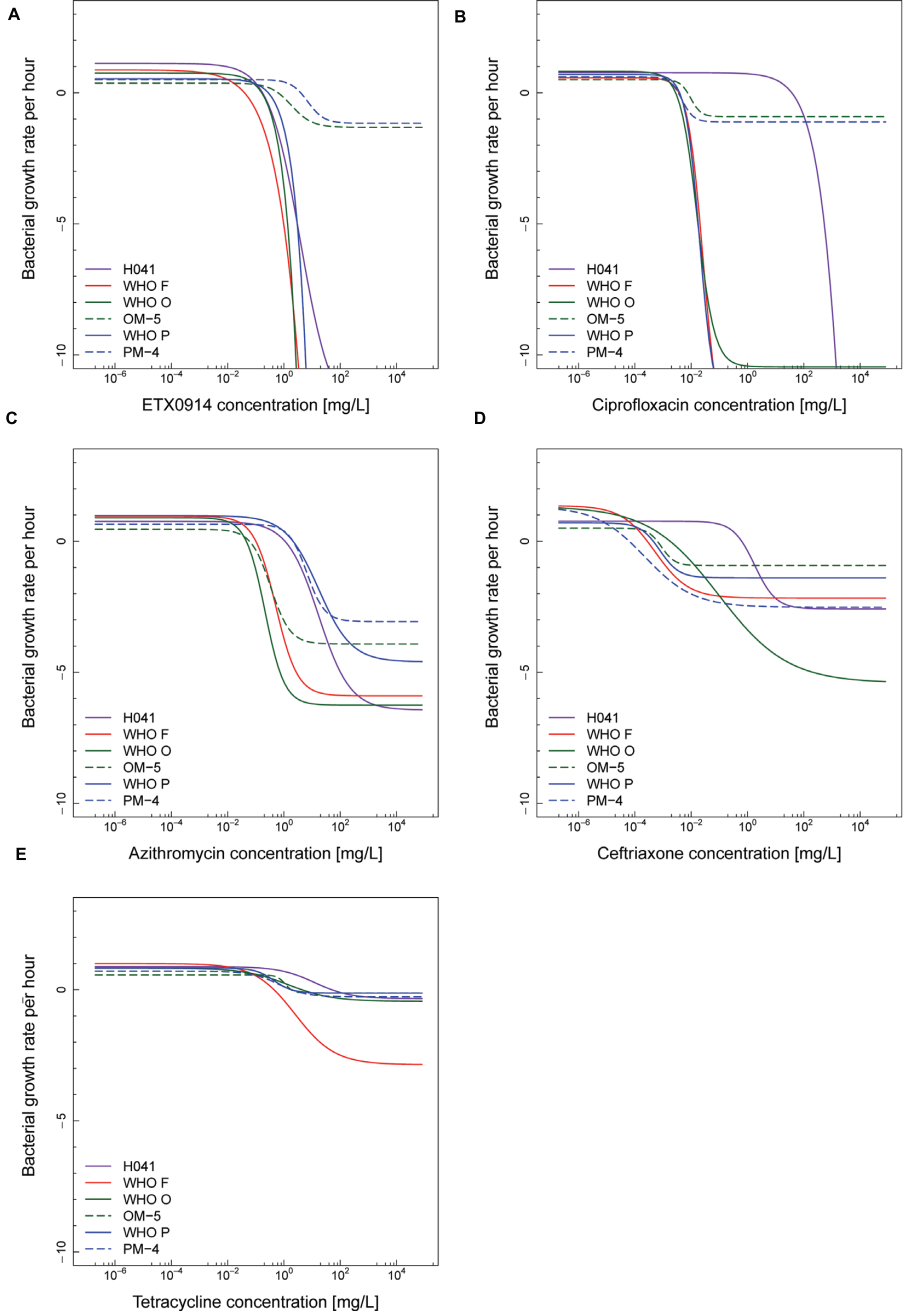
**Comparisons of pharmacodynamic functions based on the time-kill curve data for ETX0914 **(A)**, ciprofloxacin **(B)**, azithromycin **(C)**, ceftriaxone **(D)**, and tetracycline **(E)** and six different *Neisseria gonorrhoeae* strains: H041, WHO F, WHO O, OM-5 (*in vitro* selected resistant mutant from WHO O), WHO P, and PM-4 (*in vitro* selected resistant mutant from WHO P).** The bacterial growth rates per hour derived from linear regression on time-kill curves for each antimicrobial concentration are plotted.

The net growth rates for each ETX0914 concentration examined were calculated and a pharmacodynamic function fitted to the data (**Figure 2**). The minimal bacterial net growth rate at high concentrations of antimicrobial (parameter ψ_min_) was not identifiable (below ∼10 h^-1^) for all the wild type strains and was substantially higher for both the resistant mutants (-1.2 for OM-5 and -1.4 for PM-4). The steepness of the slope of the pharmacodynamic relationship (Hill coefficient; parameter κ) did not differ for the wild type strains compared to the resistant mutants. The pharmacodynamic zMIC (concentration that results in net growth rate of zero) of ETX0914 was within one MIC doubling dilution of those obtained with agar dilution technique. The pharmacodynamic parameter estimates for ETX0941 were compared to those of ciprofloxacin, azithromycin, ceftriaxone, and tetracycline (**Figure 3**). In general, each antimicrobial resulted in a unique pharmacodynamic profile. For ciprofloxacin, the “minimal growth rate” (parameter ψ_min_) was low for all four wild type strains (**Figure 3B**). For the highly ciprofloxacin-resistant strain H041, the “minimal growth rate” (ψ_min_) was below detection limit (100 CFU/mL) and did not level off in the range of examined concentrations (<-15). However, the “minimal growth rate” (ψ_min_) was significantly increased in both the resistant mutants, i.e., similar as was observed for ETX0914. The high zMIC of ciprofloxacin in H041 shifted its curve away from the curves of the susceptible strains (**Figure 3B**). Ciprofloxacin and ETX0914 had the lowest “minimal growth rates” (ψ_min_), followed by azithromycin, ceftriaxone, and tetracycline. Tetracycline exposure resulted in “minimal growth rate” (ψ_min_) values relatively close to zero in all strains with exception of WHO F (**Figure 3E**).

**FIGURE 4:**
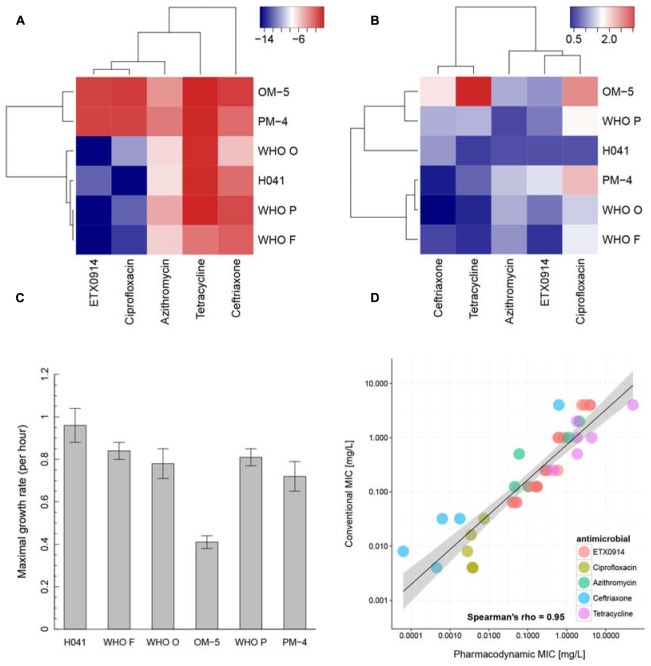
**Pharmacodynamic properties of ETX0914 in direct comparison to ciprofloxacin, azithromycin, ceftriaxone and tetracycline.** **(A)** Shows a hierarchical clustering for the minimal bacterial net growth rate at high concentrations of antimicrobial (parameter ψ_min_), **(B)** for the steepness of the slope of the pharmacodynamic function (Hill coefficient, κ), **(C)** shows the maximal growth rate in the absence of antimicrobial (parameter ψ_max_) for each strain calculated as mean and standard error from all independent experiments (**Supplementary Table S1**) and **(D)** the correlation between MICs measured with conventional methods (Etest or agar dilution technique; values below <0.002 mg/L and >32 mg/L (Etest detection limit) were excluded from the analysis) and pharmacodynamic MIC (zMIC). The linear regression line is drawn in black and the confidence interval shaded in gray. The heatmap was produced by plotting the numeric values of ψ_min_ and the Hill coefficient (κ) in a color gradient from red (high values) to blue (low values). The hierarchical clustering was performed using the complete linkage algorithm as implemented in the package heatmap3 (Zhao et al., 2014). The distances in the resulting dendrogram represented similarities between strains and antimicrobials.

In the hierarchical clustering of the “minimal growth rate” (ψ_min_) (**Figure 4A**), ETX0914 and ciprofloxacin clustered together. Azithromycin showed relatively close similarity to this cluster, followed by ceftriaxone and tetracycline. The two resistant mutants OM-5 and PM-4 clustered together and could be clearly distinguished from the wild type strains. Hierarchical clustering did not reveal any clearly distinguishable pattern between the steepness of the slope of the pharmacodynamic relationship (Hill coefficient; parameter κ) for resistant mutants and the wild type strains. Only the XDR strain H041 appeared distinctly separated from the other strains in this clustering. The highest value of the Hill coefficient (steepest slope of the pharmacodynamic relationship) were obtained for ciprofloxacin (**Figure 4B**). The maximal growth rate in the absence of antimicrobial (ψ_max_ parameter) ranged between 0.3 and 1.4 h^-1^ for the susceptible strains, corresponding to a bacterial doubling time of 30–139 min. This parameter was substantially lower in the resistant mutants compared to their respective wild type strains (**Figure 4C**). Overall, the pharmacodynamic zMIC values were very similar to the MICs determined using agar dilution technique and/or Etest (Spearman rank correlation coefficient 0.95). The 95% confidence interval for the regression line was substantially wider for the very low and high MIC values (**Figure 4D**).

### Checkerboard Assay and Time-kill Curve Analysis for Antimicrobial Combinations

The checkerboard assay identified an indifferent effect (FICI = 0.5–3) for most antimicrobial combinations and strains (**Table 4**). However, the combination of ETX0914 and ciprofloxacin indicated synergism for the resistant mutants OM-5 and PM-4 in both the checkerboard assay (FICI range: 0.4–1 for both mutants) and time-kill curve analysis (FICI: 0.1 and 0.10, respectively). The combination of ETX0914 and ciprofloxacin also indicated synergism for the wild type strain WHO F in the time-kill curve analysis (FICI: 0.4), and in the checkerboard assays the corresponding FICI was at the breakpoint for synergy, that is, 0.5. Furthermore, the combination of ETX0914 and ceftriaxone indicated synergism for the wild type WHO P strain and its isogenic selected resistant mutant (PM-4) in the time-kill curve analysis (FICI: 0.3 and 0.02, respectively), and in the checkerboard assays the corresponding FICIs were at the breakpoint for synergy, that is, 0.5–0.6. The only antagonism indicated in the checkerboard assays was for the combination of ETX0914 and tetracycline and the XDR strain H041 (FICI range: 2–8), but no antagonism was detected in the time-kill curve analysis (FICI = 1.9). A qualitative comparison of the time-kill curves revealed that ETX0914 combined with ciprofloxacin or azithromycin resulted in a more rapid bactericidal effect compared to ETX0914 alone (**Supplementary Figure S1**). For the resistant mutants, the “minimal growth rate” (ψ_min_) was significantly lower with both these combinations compared to ETX0914 alone.

**Table 4:** Fractional inhibitory concentration index (FICI) from the checkerboard assay and time-kill curve analysis.

Strain	Antimicrobial combination	Checkerboard FICI	Time-kill FICI
H041	ETX0914 + CIP	1.0–2.2	1.3
WHO F	ETX0914 + CIP	0.5–1.0	0.4
WHO O	ETX0914 + CIP	0.5–1.0	1.6
OM-5	ETX0914 + CIP	0.4–1.0	0.1
WHO P	ETX0914 + CIP	0.8–2.0	1.6
PM-4	ETX0914 + CIP	0.4–1.0	0.1
H041	ETX0914 + AZM	2.0–3.0	1.5
WHO F	ETX0914 + AZM	1.0–1.5	1.3
WHO O	ETX0914 + AZM	0.6–1.0	0.7
OM-5	ETX0914 + AZM	0.6–2.0	1.7
WHO P	ETX0914 + AZM	1.0–2.5	2.2
PM-4	ETX0914 + AZM	1.0–1.2	0.9
H041	ETX0914 + CRO	1.0–2.0	1.3
WHO F	ETX0914 + CRO	1.0–1.5	1.1
WHO O	ETX0914 + CRO	1.0–2.0	1.4
OM-5	ETX0914 + CRO	0.6–1.0	0.8
WHO P	ETX0914 + CRO	0.6–1.2	0.3
PM-4	ETX0914 + CRO	0.5–1.2	0.02
H041	ETX0914 + TET	2.0–8.0	1.9
WHO F	ETX0914 + TET	1.0–2.0	2.1
WHO O	ETX0914 + TET	1.0–1.2	0.9
OM-5	ETX0914 + TET	1.0–2.0	0.9
WHO P	ETX0914 + TET	1.0–1.5	2.3
PM-4	ETX0914 + TET	0.8–1.1	1.8


*CIP, ciprofloxacin; AZM, azithromycin; CRO, ceftriaxone; TET, tetracycline.*

## Discussion

This study extends the characterization of *N. gonorrhoeae* resistance determinants for the novel spiropyrimidinetrione ETX0914; the new D429A resistance mutation in GyrB was identified and it was shown that the MtrCDE eﬄux pump exports ETX0914 (MtrCDE inactivation restored susceptibility to wild type MIC levels). The effects of ETX0914 on the growth of *N. gonorrhoeae* (ETX0914 wild type, single step selected resistant mutants, and eﬄux pump mutants) were detailed in a novel *in vitro* time-kill curve analysis to estimate pharmacodynamic parameters (Foerster et al., 2015) of the new antimicrobial. For comparison, ciprofloxacin, azithromycin, ceftriaxone and tetracycline were also investigated, separately and in combination with ETX0914, using time-kill curve analysis and checkerboard analysis. ETX0914 was rapidly bactericidal for all wild type strains and had similar pharmacodynamic properties to ciprofloxacin. ETX0914 in combination with azithromycin and ceftriaxone was highly effective and synergistic interaction with ciprofloxacin, particularly for ETX0914-resistant mutants, was also found.

Single step ETX0914-resistant mutants, ETX0914 MICs of 0.5–4 mg/L, were selected at very low frequencies on GC agar plates (<1.9 × 10^-14^) from the wild type *N. gonorrhoeae* WHO F, O, and P strains, but not from H041. All resistant mutants had mutations in the DNA gyrase subunit GyrB. In addition to previously identified *gyrB* D429N and K450T mutations in selected ETX0914-resistant mutants (Alm et al., 2015), the present study also found a D429A mutation in one of the resistant mutants. Interestingly, using the more nutritious GCAGP agar medium (including also 1% hemoglobin and 10% horse serum) and selection during 72 h incubation, resistant mutants were obtained from all four strains and in a higher frequency, that is, with a frequency of 5 × 10^-9^ for WHO F, 1 × 10^-8^ for WHO O and WHO P, and 8 × 10^-9^ for H041, consistent with a previous publication (Alm et al., 2015). Notably, no *gyrB* mutations were found in H041. The choice of medium used for selecting mutants strongly influences the mutational frequency, which shows the difficulties of predicting how rapidly resistance to ETX0914 will be selected *in vivo*. Furthermore, in the present study it was also obvious that the resistance mutations in *gyrB* reduced the growth rates compared to their isogenic wild type strains and this decreased biological fitness might also make it difficult to predict the initial spread of ETX0914-resistant mutants *in vivo*.

Inactivation of the MtrCDE eﬄux pump increased susceptibility in wild type strains and restored the susceptibility to wild type levels in the selected ETX0914-resistant mutants (from 2–4 mg/L to 0.125 mg/L). In one strain (H041), inactivation of also the MacAB and NorM eﬄux pumps substantially decreased the MIC of ETX0914 (from 0.125 mg/L to 0.008 mg/L). This shows that eﬄux pumps, especially the MtrCDE pump, can influence susceptibility to ETX0914.

Pharmacodynamic modeling and prediction of the efficacy of different dosing regimens for cefixime and ceftriaxone have been previously performed using a combination of clinical data and *in vitro* data (Chisholm et al., 2010). There is, however, no appropriate *in vitro* method to determine whether single dose or multiple doses of new antimicrobials, such as ETX0914, in monotherapy or in dual therapy provide effective bactericidal activity. The present study quantified the antibacterial effects of ETX0914 and compared the results to those of previously or currently used antimicrobials by calculating the net growth rates from time-kill curve analysis and fitting a pharmacodynamic function to the time-kill curve data. The highest concentration included was 16 × MIC (measured with agar dilution technique prior to the experiment), to avoid toxic off-target effects that can occur at unrealistically high antimicrobial concentrations. For all wild type strains, ETX0914 exhibited bactericidal activity (>99.9% killing) after only 4 h at 8 × MIC and 16 × MIC, and more gradual bactericidal activity at 2 × MIC and 4 × MIC in the susceptible strains, but not in the mutants. Analysis of the pharmacodynamic function to predict the concentration- and time-dependent properties of antimicrobials from time-kill curve data was established in an *Escherichia coli* model (Regoes et al., 2004). For antimicrobials with a steep slope of the pharmacodynamic relationship, as characterized by a high value for the Hill coefficient (parameter κ), the C_max_/MIC ratio should be maximized, showing concentration dependency. When MIC, steepness of the slope of the pharmacodynamic function (Hill coefficient; parameter κ) and maximal bacterial net growth rate in the absence of antimicrobial (ψ_max_) were equal, low values of the minimal bacterial net growth rate at high concentrations of antimicrobial (ψ_min_) were suggested to predict efficacy of the antimicrobial. That study, however, only examined a single *E. coli* strain over a time-course of 70 min (Regoes et al., 2004). The present study examined the parameters of the pharmacodynamic function for several different *N. gonorrhoeae* strains (wild type and selected ETX0914-resistant mutants) and an extended 6 h sampling window. An excellent correlation between the zMIC and the MIC measured with Etest or agar dilution technique was found, despite the different growth medium and exposure time. This indicates that the time-kill curve analysis was optimized, quality assured and consistent. The highest deviations were found for ceftriaxone, probably because the sampling period of 6 h was too short to capture the full effect of this time-dependent antimicrobial.

Hierarchical clustering also helped to find similarities across antimicrobials and strains. The “minimal growth rate” (ψ_min_) summarized the highest killing rate measured within the 6 h assay time in response to the antimicrobial. The values for the “minimal growth rate” (ψ_min_) were much higher in the isogenic ETX0914-resistant mutants compared to the susceptible wild type strains and these clustered together. Nevertheless, additional work is needed to evaluate whether this increase in the “minimal growth rate” (ψ_min_) is characteristic of *gyrB* mutations or if it is generalizable to other resistance mechanisms and antimicrobials. In general, ETX0914 and ciprofloxacin showed the highest similarity in the clustering across all strains, which is not surprising considering that both compounds target the same enzyme(s) and are mechanistically quite similar. These similarities are not captured by means of conventional MIC determinations alone. The time-kill curve analysis and subsequent pharmacodynamic analyses used in this study provide additional information. The hierarchical clustering of values of the steepness of the slope of the pharmacodynamic function (Hill coefficient; parameter κ) resulted in a less clear pattern and the Hill coefficient (κ) differed greatly between strains but not significantly in the mutants compared to the wild type strains. There was a non-significant trend for steeper slopes of the pharmacodynamic functions [higher Hill coefficient (κ) values] in the time-dependent antimicrobial ceftriaxone and the bacteriostatic compound tetracycline compared to the more bactericidal azithromycin, ETX0914 and ciprofloxacin.

The results of *in vitro* time-kill curve analysis and pharmacodynamic functions for ciprofloxacin, azithromycin, ceftriaxone, and tetracycline were, in general, consistent with previous findings using other pharmacodynamic analysis methods (Drusano, 2004). For drugs like fluoroquinolones (e.g., ciprofloxacin) and macrolides (e.g., azithromycin), a high ratio of maximum concentration to MIC (*C*_max_/MIC ratio) predicts a successful treatment outcome (Li et al., 1999; Drusano, 2004). This requires maximizing the dose given to a patient. ETX0914 was rapidly bactericidal with a pharmacodynamic profile similar to ciprofloxacin, so maximizing the concentration of ETX0914 in a single dose should be highly effective. In contrast, for time-dependent drugs (including β-lactams and tetracyclines) a long time of free antimicrobial above MIC (f*T*/MIC ratio) or a large ratio of the area under the pharmacokinetic curve to MIC (AUC/MIC) predicts a successful treatment outcome. Therefore, several smaller doses or continuous infusions to maximize the time above MIC can have a better treatment outcome than one large dose (Jacobs, 2001; Drusano, 2004; Asín-Prieto et al., 2015).

Combinations of ETX0914 with ciprofloxacin, azithromycin, ceftriaxone, or tetracycline were explored comparing FICIs from the time-kill curve analysis and checkerboard assay. In general, the results of the time-kill curve analysis and checkerboard assay were concordant, but the error for estimating the zMIC was high in some pharmacodynamic models and this was reflected in the uncertainty of the FICI values. ETX0914 and ciprofloxacin showed synergy particularly in the ETX0914 resistant mutants. It was also recently found that specific *gyrB* mutations might result in a conformational change creating a more favorable binding pocket for ciprofloxacin and, accordingly, a decreased ciprofloxacin MIC in ciprofloxacin-resistant *N. gonorrhoeae* strains (Alm et al., 2015) as well as the fact that K450T mutation, resulting in ETX0914 resistance, conveys increased susceptibility to ciprofloxacin (Kern et al., 2015). The combination of ETX0914 and ciprofloxacin might be beneficial because ETX0914 acts bactericidal on ciprofloxacin resistant strains and vice versa. The drug interaction between ETX0914 and ciprofloxacin strengthens the hypothesis of two different targets that influence each other. Non-competitive inhibition of the same enzyme by two antimicrobials that follow the rule of Bliss independence will also lead to synergy in a theoretical model based on Michaelis Menten kinetics (Breitinger, 2012). The combination of ETX0914 with azithromycin showed a more rapid killing in a qualitative analysis of the time-kill curve and no antagonism was observed for any of the examined strains in the time-kill curve analysis or checkerboard assays. These results indicate that ETX0914 could also be beneficially administered together with azithromycin in an oral dual antimicrobial therapy, which also would be able to treat concomitant sexually transmitted infections such as *Chlamydia trachomatis* and many *Mycoplasma genitalium* infections.

## Conclusion

ETX0914 (AZD0914) was rapidly bactericidal and had similar pharmacodynamic properties to ciprofloxacin. Specific mutations in GyrB result in resistance to ETX0914 and MtrCDE inactivation restore susceptibility to wild type levels. ETX0914 alone and in dual combinations with ciprofloxacin, azithromycin, or ceftriaxone were highly effective against *N. gonorrhoeae*. ETX0914, as oral monotherapy or combined with azithromycin (to cover also additional sexually transmitted infections), should be considered for appropriate Phase III clinical trials and future gonorrhea treatment.

## Author Contributions

SF, NL, CLA, WS, and MU designed and initiated the study. SF, DG, SJ, LH, and MU coordinated and performed all the laboratory analyses. SF and MU analyzed and interpreted all the data, and wrote a first draft of the paper. All authors read, commented on and approved the final manuscript.

## Transparency Declaration

WS is a recipient of a Senior Research Career Scientist Award from the Biomedical Laboratory Research and Development branch of the Medical Research Service of the United States Department of Veterans Affairs. The contents of the paper do not represent the views of the Department of Veterans Affairs or the United States government.

## Conflict of Interest Statement

The authors declare that the research was conducted in the absence of any commercial or financial relationships that could be construed as a potential conflict of interest.
