# Supplementary material for: Genetic Resistance Determinants, In Vitro Time-Kill Curve Analysis and Pharmacodynamic Functions for the Novel Topoisomerase II Inhibitor ETX0914 (AZD0914) in Neisseria gonorrhoeae
Source: Front Microbiol. 2015 Dec 10;6:1377. doi: 10.3389/fmicb.2015.01377 (PMC4674575; doi:10.3389/fmicb.2015.01377)
Supplement: Supplementary file 2 [file Image_1.PDF]

Supplementary Material

Genetic resistance determinants, *in vitro* time-kill curve analysis and pharmacodynamic functions for the novel topoisomerase II inhibitor ETX0914 (AZD0914) in *Neisseria gonorrhoeae*

Sunniva Förster[1,2,3], Daniel Golparian[3], Susanne Jacobsson[3], Lucy J. Hathaway[1], Nicola Low[2], William M. Shafer[4,5], Christian L. Althaus[2] and Magnus Unemo[3]*

*** Correspondence:**

Magnus Unemo, Örebro University Hospital, Department of Laboratory Medicine, Clinical Microbiology, SE-70185 Örebro, Sweden

e-mail: magnus.unemo@regionorebrolan.se

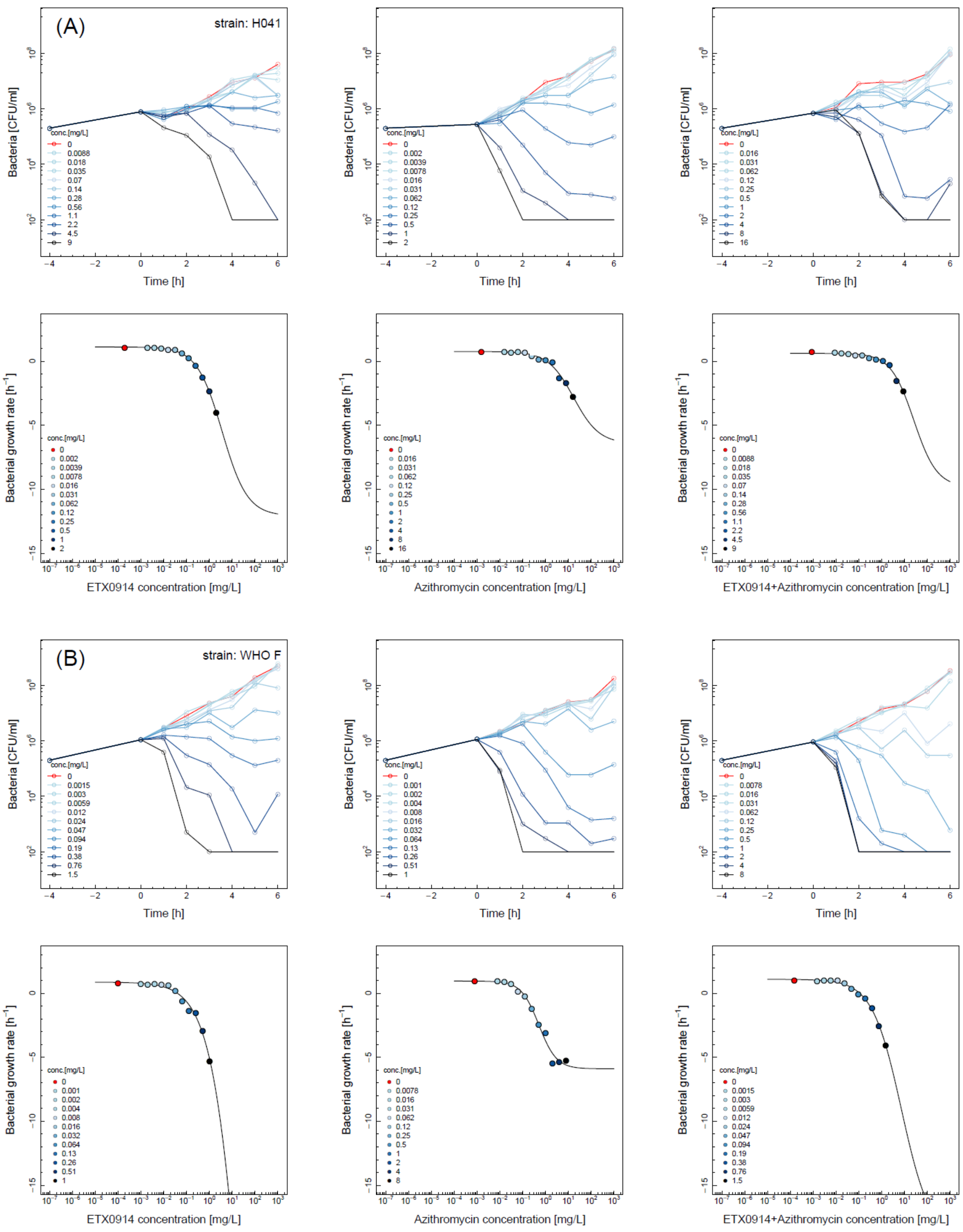
(A)
strain: H041
Bacteria [CFU/ml]
Time [h]
Bacterial growth rate [h⁻¹]
ETX0914 concentration [mg/L]
Azithromycin concentration [mg/L]
ETX0914+Azithromycin concentration [mg/L]
(B)
strain: WHO F
conc.[mg/L]

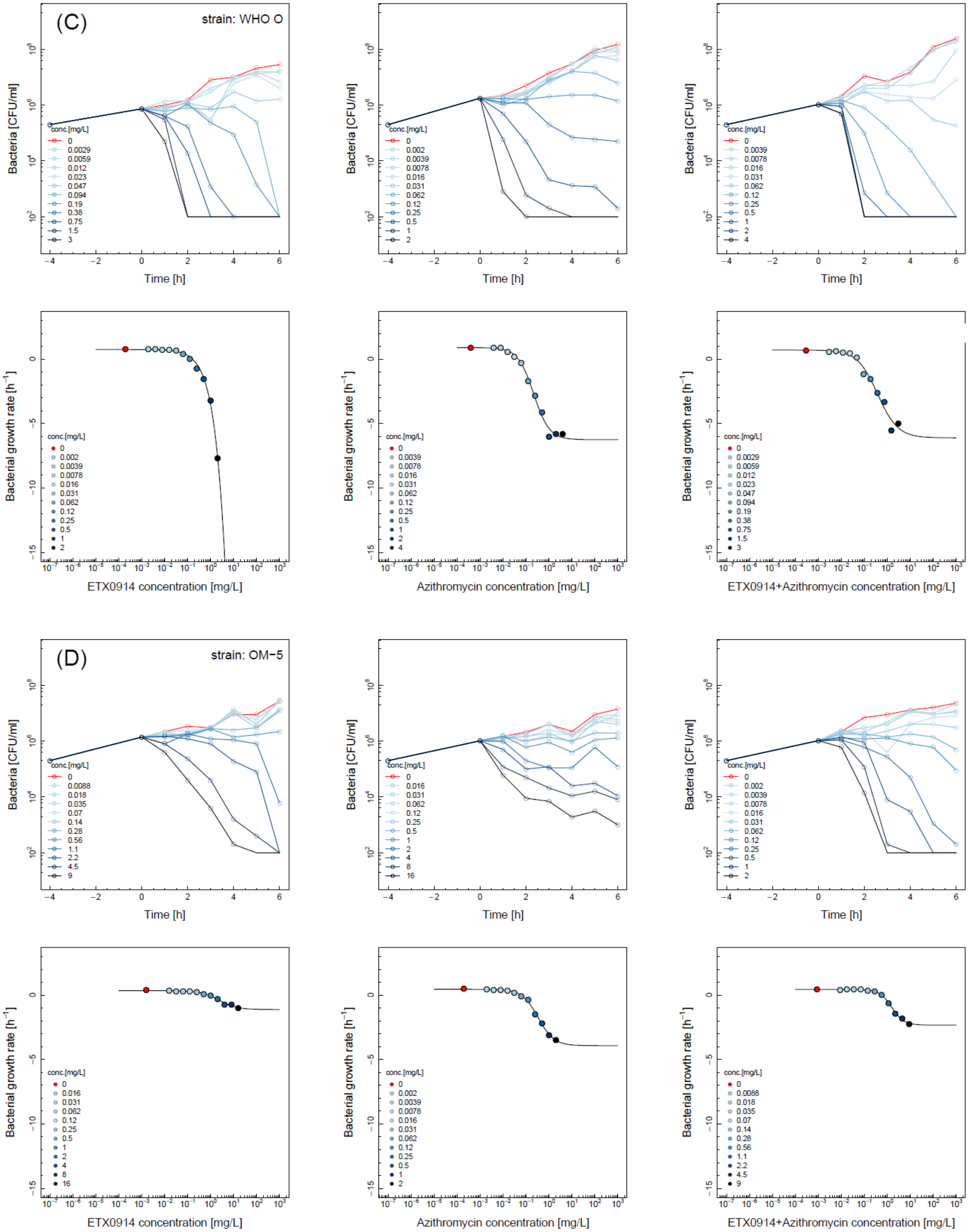
(C)
strain: WHO O
Bacteria [CFU/ml]
Time [h]
conc.[mg/L]
0
0.0029
0.0059
0.012
0.023
0.047
0.094
0.19
0.38
0.75
1.5
3
conc.[mg/L]
0
0.002
0.0039
0.0078
0.016
0.031
0.062
0.12
0.25
0.5
1
2
conc.[mg/L]
0
0.0039
0.0078
0.016
0.031
0.062
0.12
0.25
0.5
1
2
4
Bacterial growth rate [h⁻¹]
ETX0914 concentration [mg/L]
Azithromycin concentration [mg/L]
ETX0914+Azithromycin concentration [mg/L]
(D)
strain: OM−5
Bacteria [CFU/ml]
Time [h]
conc.[mg/L]
0
0.0088
0.018
0.035
0.07
0.14
0.28
0.56
1.1
2.2
4.5
9
conc.[mg/L]
0
0.016
0.031
0.062
0.12
0.25
0.5
1
2
4
8
16
conc.[mg/L]
0
0.002
0.0039
0.0078
0.016
0.031
0.062
0.12
0.25
0.5
1
2
Bacterial growth rate [h⁻¹]
ETX0914 concentration [mg/L]
Azithromycin concentration [mg/L]
ETX0914+Azithromycin concentration [mg/L]

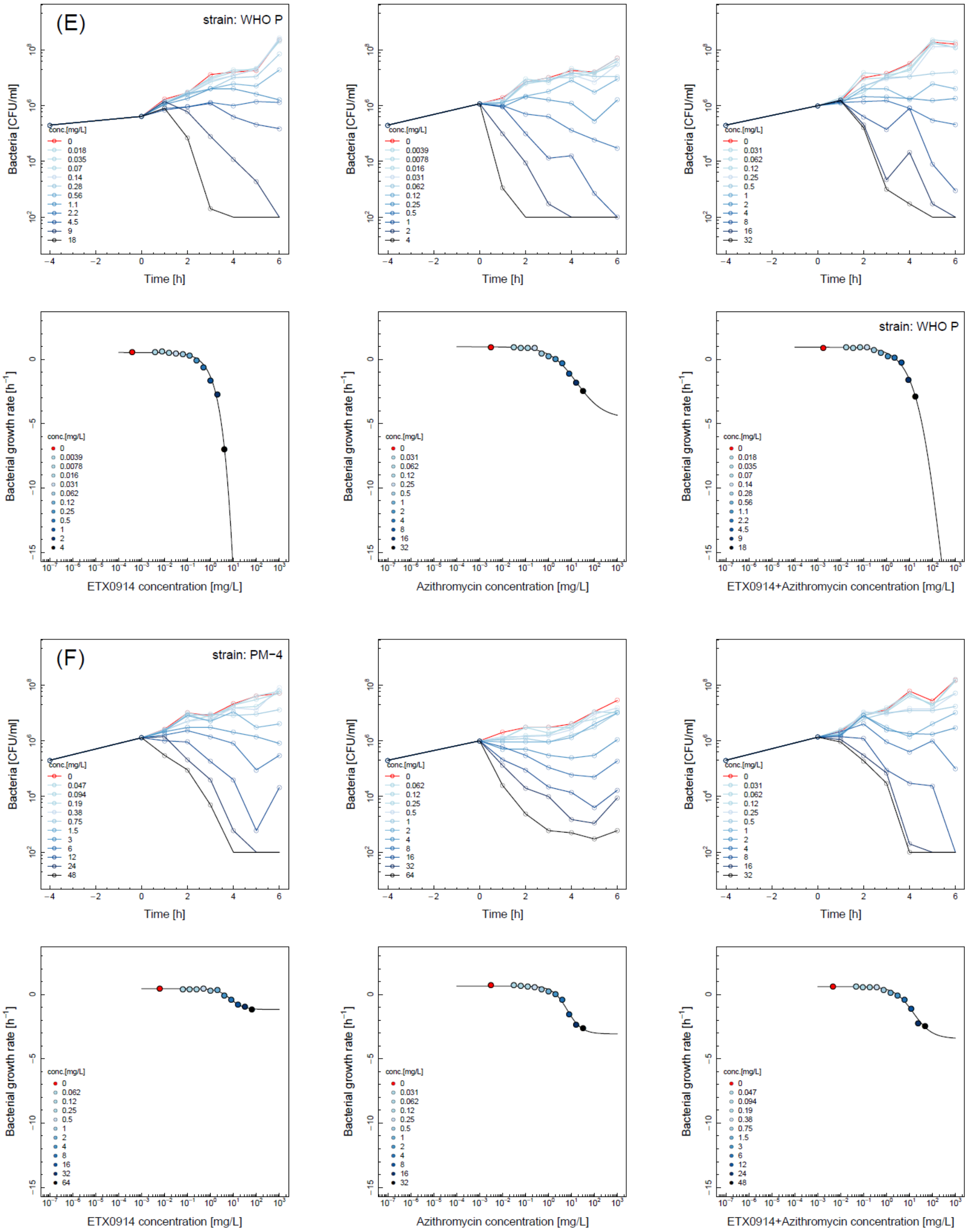
(E)
strain: WHO P
Bacteria [CFU/ml]
Time [h]
conc.[mg/L]
0 0.018 0.035 0.07 0.14 0.28 0.56 1.1 2.2 4.5 9 18
conc.[mg/L]
0 0.0039 0.0078 0.016 0.031 0.062 0.12 0.25 0.5 1 2 4
conc.[mg/L]
0 0.031 0.062 0.12 0.25 0.5 1 2 4 8 16 32
Bacterial growth rate [h⁻¹]
conc.[mg/L]
0 0.0039 0.0078 0.016 0.031 0.062 0.12 0.25 0.5 1 2 4
ETX0914 concentration [mg/L]
conc.[mg/L]
0 0.031 0.062 0.12 0.25 0.5 1 2 4 8 16 32
Azithromycin concentration [mg/L]
strain: WHO P
conc.[mg/L]
0 0.018 0.035 0.07 0.14 0.28 0.56 1.1 2.2 4.5 9 18
ETX0914+Azithromycin concentration [mg/L]
(F)
strain: PM−4
Bacteria [CFU/ml]
Time [h]
conc.[mg/L]
0 0.047 0.094 0.19 0.38 0.75 1.5 3 6 12 24 48
conc.[mg/L]
0 0.062 0.12 0.25 0.5 1 2 4 8 16 32 64
conc.[mg/L]
0 0.031 0.062 0.12 0.25 0.5 1 2 4 8 16 32
Bacterial growth rate [h⁻¹]
conc.[mg/L]
0 0.062 0.12 0.25 0.5 1 2 4 8 16 32 64
ETX0914 concentration [mg/L]
conc.[mg/L]
0 0.031 0.062 0.12 0.25 0.5 1 2 4 8 16 32
Azithromycin concentration [mg/L]
conc.[mg/L]
0 0.047 0.094 0.19 0.38 0.75 1.5 3 6 12 24 48
ETX0914+Azithromycin concentration [mg/L]

Supplementary Figure 1. Qualitative comparison of antimicrobial combination in six different *Neisseria gonorrhoeae* strains H041 (A), WHO F (B), WHO O (C), OM-5 (D; *in vitro* selected resistant mutant from WHO O), WHO P (E), and PM-4 (F; *in vitro* selected resistant mutant from WHO P). The upper panel shows time-kill curves and the lower panel pharmacodynamics functions. The single treatment ETX0914 (left) and azithromycin (middle) is compared to the combination of ETX0914 and azithromycin (right) in 50% ratios.
